# The Teeth from a Medico-Legal Aspect

**Published:** 1883-09

**Authors:** 


ARTICE VIII.

### THE TEETH FROM A MEDICO-LEGAL ASPECT.

From a Medico-legal aspect the teeth have not received the attention to which their importance, in many particulars, entitles them. It is, indeed, astonishing to find how seldom they are referred to in standard works on Forensic Medicine; even that much appreciated and consulted work of Casper's does not, so far as we can gather, allude to them. Yet that they have an importance in such matters is evident from the information they have furnished from time to time.

One of the earliest appeals to this portion of the human frame was as a test of age. About the year 1836, our Legislature was engaged in amending an Act which was designed to limit the age of children employed in factories, and as physical development was known to be a very uncertain criterion of age, some better test, to prevent the evasion of the Act, was anxiously sought for.

To a member of our own body, Mr. E. Saunders, belongs the credit of having first shown that within certain periods of life,. the teeth may be relied on as affording evidence of age. This gentleman undertook the collection of a large number of statistics derived from the inspection of the mouths of children.

The data thus obtained by Mr. Saunders, and published as a monograph in 1837, entitled, "The Teeth, a test of Age," were generally confirmed by the collection of the results of a very much larger number of examinations conducted by the present Mr. S. Cartwright. From the tables founded upon these observations the ages of persons varying from six to fourteen years can be pretty correctly ascertained.

But the teeth afford valuable evidence of age at other periods of life as well, though the fact has been much neglected. Thus whilst Casper gives some excellent data, embodied in tables, for ascertaining the age of the fœtus and newly-born child, these are derived from the development of the bones and organs other than the teeth, yet the researches of J. Tomes, Legros and Magitot, and others, n this direction, have added to our knowledge facts which would afford most valuable assistance in such investigations. Again the periods occupied in the eruption of the temporary teeth are now, for the average, pretty well ascertained, whilst the later researches of Magitot enable us to fill up the gap between the completion of the first, and the commencement of the second dentition.

In another point of view the teeth may serve to afford evidence useful in Medico-legal investigation. For instance, there are the now well recognized syphilitic teeth, which may hereafter occupy no mean place in the dicisions of divorce courts.

But the teeth doubtless are of more importance in the matter of identification than in any other, and it is surprising how little this line of inquiry has been pursued. In a few cases suggestions of their value under the above conditions have been offered. In one of the most important trials ever recorded, viz: that of the personation of a supposed dead person, both counsel for plaintiff and defendant were advised that much valuable information could be obtained by an examination of the mouth; the former by the dentist who had attended the individual supposed to be dead, and who confidently stated that he could recognize the true man by his teeth, but that counsel, for good reasons no doubt, declined such evidence. In the matter of the lamented Prince Imperial, whose life was sacrificed in Zululand, when a doubt was raised as to the identification of his remains, two eminent dentists who had operated upon his teeth offered to give, if necessary, information which would have been conclusive.

It was under this phase of the question that Mr. Turner at the last meeting of the Odontological Society exhibited to the members casts of both jaws, also the lower jaw itself, of the unfortunate victim of a presumed murder. The information afforded by the models and maxilla pointed to the age as being about fifteen. From the development of the third molars Mr. Hutchinson suggested a rather later age, but the small amount of wear the teeth had undergone in the opinion of Mr. Turner, Mr. C. S. Tomes, and Mr. Coleman led them to fix the age at between fourteen and sixteen. A singular circumstance was that the two lower second temporary molars were retained, their successors being just below them, and this might, under certain conditions have led to an identification of the unfortunate being. Both Mr. Turner and Mr. C. S. Tomes strongly insisted on the greater value the teeth were able to afford in Medico-legal questions than had hitherto been assigned to them.
—*Journal of British Dental Association.*

